# How employees of Korean franchise restaurants perceive older adult kiosk accessibility

**DOI:** 10.3389/fpubh.2025.1660819

**Published:** 2026-01-08

**Authors:** Chaeyun Jang, Seongcheol Kim

**Affiliations:** College of Media and Communication, Korea University, Seoul, Republic of Korea

**Keywords:** digital accessibility, digital inclusion for the older adults, kiosk, restaurant industry in South Korea, social representation theory

## Abstract

This study examines how employees at franchise restaurants in South Korea perceive the accessibility of kiosks, which are increasingly replacing face-to-face service in the restaurant industry. While kiosks can enhance operational efficiency and reduce labor costs, they may also marginalize older adults, who often face barriers in digital environments. In a super-aged society like South Korea, ensuring equitable access to everyday services has become a critical social issue. To address this challenge, this study uses Social Representation Theory (SRT) to examine how employees in the industry interpret and respond to accessibility issues faced by older adult users. Data was collected through semi-structured interviews with 12 employees working in franchise restaurant operations. Through content and core–periphery analyses, this study identified two overarching perceptions: employees view improving older adult kiosk accessibility as a business opportunity and an organizational and social challenge. While participants recognized opportunities such as reduced labor costs and enhanced convenience for owners and customers, they also highlighted substantial difficulties in making kiosks more accessible for older adult users. These challenges include operational burdens and tensions between profitability and accessibility efforts. This study emphasizes that understanding corporate operators’ shared perceptions is essential for addressing older adult kiosk accessibility. Doing so enables private-sector organizations to contribute to digital inclusion while aligning social value creation with business sustainability.

## 1 Introduction

Digital transformation (DT) is defined as a comprehensive change encompassing not only technological advancements but also modifications in customer experience and operational processes (1). Traditionally, the restaurant industry has been a service-oriented sector, relying heavily on face-to-face interactions and emphasizing customer service and human engagement rather than facility-centric operations (2). However, in response to evolving consumer lifestyles and demands, Korea’s restaurant industry has been increasingly expanding its non-face-to-face services and embracing digital transformation across its operations. This transformation comprises adopting new technologies and substantially changing organizational structures, workforce operations, and the customer experience. In this process, digital alternatives are replacing traditional services.

Increasingly, kiosks are the primary interface between customers and service points. A kiosk is a touchscreen system designed for information delivery and unattended payment processing, functioning as a technology-based, self-service system that replaces human labor (3). In Korea’s restaurant industry, businesses that sell foods such as pizza and hamburgers, and other fast-food establishments, recorded the second-highest Korea Restaurant Business Index (KRBI) scores in early 2025, following only institutional cafeterias (4). Experts interpret these trends as resulting from ongoing economic pressures, including high food prices and labor shortages, which are reshaping the restaurant industry. As a result, adopting kiosks is no longer viewed as an optional strategy but as a necessary one for maintaining operational efficiency. According to a recent survey, 93.8% of respondents from small- and medium-sized enterprises that had adopted kiosks reported that the kiosks had enhanced business operations, particularly through reduced labor costs (5).

Kiosks have offered customers the convenience of unattended ordering, thus improving operational efficiency and restaurant industry performance, particularly concerning labor cost reduction. However, as kiosks replace face-to-face services, customers with lower digital literacy levels face challenges and potential exclusion from digital technology. According to the OECD’s Report on Inclusive Growth, digital transformation cannot be considered fully positive or inclusive unless all individuals can participate in and benefit from it (6). Therefore, ensuring digital accessibility for all users is a critical issue; it must be addressed alongside technological advancements.

Nevertheless, older adults are particularly vulnerable to digital exclusion amid accelerating digital transformations in daily life. Prior research has already shown that cognitive decline, lower levels of digital literacy, and social pressure contribute to negative experiences for older adult users when interacting with kiosks (7). This study also highlights that digital exclusion, particularly regarding kiosk usage, may lead to the older adults being excluded from essential services that could otherwise help alleviate their economic and social challenges. Given the crucial role kiosks play in everyday activities such as placing orders and making payments, there is a growing and imperative need to develop strategies for improving accessibility for older adult users. This issue is particularly urgent in South Korea, where an aging population and declining birthrate suggest that the country will become a “super-aging society” within the next few decades (8). The lack of digital literacy and access to technology among older adults significantly affects their participation in digital society; this leads to negative experiences associated with aging, including loneliness, isolation, and feelings of uselessness (9).

In response to these pressing concerns, South Korea has established guidelines for kiosk accessibility standards in the public sector to bridge the digital divide. However, approaches that emphasize social responsibility or impose obligations on private firms have insufficiently addressed the issue. Furthermore, the inability to use technology meaningfully is not attributable solely to technical or psychological limitations; rather, individuals’ participation in information and communication technology (ICT) is shaped by social, psychological, economic, and pragmatic factors (10).

Previous research on kiosk accessibility for older adults has been conducted primarily from a user-centered perspective, focusing on device- or design-related improvements (11), including technical acceptance factors based on the UX stage to enhance user experience (12), streamlined ordering processes that reduce option complexity and increase convenience (13), improved interface readability and easy-to-use content for older adult users (14), and hardware interface enhancements such as adjusted viewing angles that accommodate older adult users’ body dimensions and arm movement range (15). Additionally, more studies have focused on kiosk accessibility in public services than in private-sector applications (16). Prior research has examined the challenges associated with self-service technology (SST) in e-government and administrative service contexts (17–19). As accessibility to administrative services is considered a fundamental right, initial kiosk research in the public sector primarily emphasized digital inclusion.

However, there is limited research on how business practitioners perceive and understand this issue within the private sector, where practical solutions must be implemented to ensure inclusion. From the franchise operator’s perspective, kiosk accessibility is a challenge that must be addressed to enhance operational efficiency, expand customer reach, and improve the overall customer experience. Self-service technology (SST) in the restaurant industry is designed not only to improve business performance but also to facilitate customer interaction by enabling managers to provide personalized SST experiences that more effectively meet consumer needs (20). However, the effective operation of SSTs presents a significant challenge for franchise operators (21).

While discussions on kiosk accessibility for older adults have largely emphasized the public sector and social value, it is crucial to recognize that private businesses are the primary entities introducing and operating kiosks in the restaurant industry. Without their active participation, achieving sustainable digital inclusion is not feasible (22). Therefore, this study underscores understanding how private-sector practitioners perceive the issue of kiosk accessibility for older individuals.

Accordingly, this study investigates how Korean franchise restaurant employees understand kiosk accessibility for older adults. This approach extends the theoretical application of SRT to the restaurant industry by providing a framework for analyzing practitioners’ shared perceptions of kiosk accessibility. It also addresses a gap in prior research, which has focused largely on user adoption or innovation resistance. By reframing older adult kiosk accessibility as an organizational challenge embedded in the digital transformation process—rather than an individual- or device-level issue—the study offers a foundation for advancing future research on digital accessibility within industry settings.

This paper is structured as follows. Section 2 reviews prior research on digital transformation in the restaurant industry, kiosk accessibility, and digital inclusion, and introduces Social Representation Theory (SRT). An outline of the methodology, which includes semi-structured interviews and core–periphery analysis, is detailed in section 3. In the next section, the results derived from the core–periphery analysis of social representations are put forward. In the penultimate section, the research findings are discussed by examining the opportunities and challenges embedded in employees’ perceptions of the intersection of kiosk and older adult accessibility. Finally, section 6 addresses the academic and practical implications, as well as the study’s limitations.

## 2 Literature review

### 2.1 The restaurant industry’s digital transformation

Digital transformation (DT) is high on many organizations’ agendas as they seek to innovate in a changing industry landscape. DT refers to a set of strategic innovations that adopt technologies to reinvent value in different organizations and at varying levels (23). As such, DT engenders a fundamental change, one that reimagines products and services as digitally enabled assets; notably, it also creates an ecosystem that enables it (24). While similar terms include digitization, that is, converting analog information to digital, and digitalization, defined as using digitized information innovatively to create and harvest value (25), DT is defined more integrally than technological change. Accordingly, DT is not limited to implementing IT solutions. Rather, it must be considered in a broader context of organizational change, cultural transformation, and a shift to a customer-centric approach (1).

In this context, COVID-19 accelerated many industries’ DT. While DT presents both opportunities and challenges, because of the pandemic, industries faced the imperative to innovate through digitalization or be left behind (26). In particular, the restaurant industry has experienced significant revenue losses, and in response, low-contact services have become commonplace. Investments have been made in companies’ digital resources to devise new processes for low-contact interactions with customers, from ordering to pick-up or delivery (27). As part of this transformation, kiosks have been adopted as an alternative to in-person ordering processes, as they can reduce interpersonal contact, lower labor costs, and streamline ordering. In the process, kiosks have been an effective tool for dealing with customers and also played an important role in customer return visits and retention (21). This has led additional restaurant companies to consider developing and implementing kiosks.

As a result, customer interactions are becoming digitalized, and there is much discussion about how digital technologies can be leveraged to enhance service delivery. Digital services are not only undergoing a physical transformation but are also evolving with the customer experience, which is increasingly personalized, interactive, and human-centric (28). This stems from the idea that organizations’ DT can fundamentally change society. High on the agenda in these discussions is the question of whether digital technologies can actually replace humans and make human efforts unnecessary across society (29).

Prior research has already shown that DT’s impact is assessed primarily at the organizational level. While organizations expect to improve operational efficiency or reduce costs through DT, the process actually increases operating costs due to fixed investments in digital resources such as infrastructure (30). Therefore, DT is about both technology and organizational and strategic transformation. Moreover, the complexity of its benefits and costs can lead to uncertain organizational performance. In addition, the changes brought about by DT can sometimes be met with resistance from multiple stakeholders, and addressing these challenges must be considered during the DT process (31). This also relates to the challenges inherent in digital inclusion efforts, whereby companies strive to make digitization accessible to more people during DT.

### 2.2 Kiosk accessibility and digital inclusion for older adults

As dependence on digital tools increases, the gap between individuals with digital skills and access and those without them is likely to widen, often along socioeconomic lines (32). Given that ICT use is now prioritized across nearly all sectors of society, the digital divide has the potential to exacerbate existing inequalities among marginalized groups. Alhassan and Adam (33) found that digital inclusion and ICT accessibility significantly influence individuals’ quality of life (QoL) on a global scale, with ICT use serving as a mediating factor in this relationship. Parsons and Hick (34) further argue that strengthening digital inclusion requires a broader agenda that examines the role of ICT in shaping social and economic participation, rather than focusing narrowly on technical accessibility alone.

The kiosk transformation of face-to-face ordering services is part of a broader DT. Digital accessibility focuses primarily on designing systems to optimize access, while inclusion involves reducing and overcoming barriers to provide equal access and opportunities for everyone (35). Therefore, digital accessibility can be seen as a transitional step toward achieving digital inclusion (36).

It is one thing for an individual to decline using digital technologies, but it is another to be excluded from digital services due to a lack of access to technology (37). Older adults are a particularly vulnerable group to digital exclusion. As the world is aging rapidly and older individuals constitute the largest group of un- or less-informed people, there are concerns about their social exclusion during DT (38). While kiosks’ rapid proliferation in the private industry is relatively recent, studies have explored their use in public organizations, banks, healthcare institutions, and other sectors.

In the early stages, kiosks were introduced primarily within the public sector as tools enabling government agencies and municipalities to communicate more effectively with citizens and to address aspects of the digital divide. Slack and Rowley (17) identified key challenges related to providing e-government services through kiosks. Traunmüller and Lenk (18) also emphasized that rapid technological development in public organizations requires addressing issues such as information security and equitable access, noting that the technological orientation of kiosk systems may hinder citizens, particularly those unfamiliar with administrative processes from locating the information they need. These studies highlight the need for improved information accessibility in public administration. According to Holgersson and Sã (39), to reduce digital exclusion and increase digital inclusion among the older adults in the DT of public services, it is essential to consider older individuals’ physical limitations, reduce their fears of using digital technologies, and demonstrate these technologies’ benefits to them.

From the user perspective, prior research has typically examined usability issues and the physical, cognitive, or experiential barriers faced by older adults. Research on technology adoption among older adults has largely aimed at proposing device or interface design guidelines that respond to age-related physical and cognitive changes (40). Another important dimension of digital accessibility involves device-level characteristics, including both hardware and software. When designing, developing, or delivering technology for older adults, understanding their needs and requirements is essential (41). Carpenter and Buday (42) emphasized ease-of-use factors such as font size, color and contrast, avoidance of technical terminology, graphical aids, speech recognition, and specialized equipment. While technological innovations offer improved convenience and various benefits, building trust in both the system and the service provider is crucial to facilitate user acceptance (43). Interface design is also a critical factor, particularly in kiosk environments where users directly interact with the machine. In kiosks, the interface is a function that consumers directly interact with when using the kiosk, and an attractive interface can make a good impression on first-time users (44).

Also, Maguire (16) aimed to identify user reactions to kiosks by analyzing prototype versions at different developmental stages. Notably, older users have been categorized as having less universal access to new kiosk technology, prompting researchers to explore this characteristic of older adults’ relationship to emerging technologies. Gao and Sun (45) investigated the usability issues of touch screens for both older and younger individuals, emphasizing the significance of implementing kiosk interfaces tailored to older users. This approach ensures their access to services, a crucial factor for enhancing older adults’ quality of life.

In addition, studies have delved into the factors influencing users’ intention to adopt kiosk technology and their usage behavior. Na et al. (46) identified that higher perceived value, social influence, performance expectancy, and hedonic motivation increase the intention to use kiosks among fast-food customers. In yet another recent investigation (7), highlighted the exclusion of older adults with relatively low levels of digital literacy from self-service technologies (SSTs) designed to replace human labor. The study revealed that perceived time pressure significantly predicted social anxiety and helplessness among older adults in kiosk interactions. Older people can struggle when using kiosks, which can cause psychological distress.

### 2.3 Kiosk in south Korean restaurant industry

Kiosks are being increasingly integrated across industries to streamline business processes, enhance operational efficiency and improve customer engagement. The self-service kiosk market is projected to grow by USD 13.28 billion between 2024 and 2029, with an estimated compound annual growth rate (CAGR) of 15.4% (47). In Korea, kiosks provide consumers with convenient access to streamlined services (48), and the adoption of unmanned systems has increased rapidly. Kiosk installations in Korea’s private sector have expanded significantly due to rising labor costs, the growth of contactless consumption and the effects of the pandemic. Since 2021, the number of kiosks installed in the country’s private sector has more than tripled compared to 2019, with a 4.1-fold increase in the food service and convenience sectors (49). The pandemic also significantly impacted on the food and beverage restaurant industry, and the reduction of physical contact among people and the shift to contactless purchases and services have indeed been transformative (50). Consequently, many traditional face-to-face service interactions are being replaced by kiosks.

From an operational perspective, the shift toward unmanned ordering systems is largely driven by rising labor costs. Although the average revenue of Korean food service businesses has continued to rise, their operating profit margins have declined from 15% in 2019 to 8.9% in 2023 due largely to increasing labor expenses (51). Among franchise restaurants, labor cost reduction was identified as the primary motivation for adopting kiosks, cited by 71.7% of franchise owners (52). As a result, the Korean food service industry has increasingly adopted not only kiosks but also mobile ordering via customers’ smartphones and tablet-based ordering systems (51). Self-service technologies (SSTs) in the restaurant industry are designed to improve operational efficiency while supporting more personalized customer interactions (20). Nevertheless, achieving strong business performance through SSTs and ensuring consistently positive customer experiences remain significant challenges for firms (21).

### 2.4 Social representation theory

Social representation theory (SRT) can be applied to examine shared ideas and beliefs about social objects (53). SRT posits that members of societies and groups form common knowledge about social objects by reconstructing shared knowledge (54). As such, individuals are social beings based in communities, and SRT seeks to understand these members’ collective knowledge. Specifically, social representational structures consist of core and peripheral concepts that stem from a set of information or opinions about an object. When different people think about the same object, the ideas presented are not all equal; instead, they are divided into a core idea and then peripheral ideas that are shared with others. It is through the central core idea that the other elements acquire concrete meaning, and the core functions to integrate and express the relationships they hold with each other (55).

SRT suggests that different social groups may perceive the same object differently based on their different collective values and objectives; thus, it can also contribute to understanding the perspectives of individuals embedded within work communities (56). In this study, SRT was applied to understand what collective and shared understandings, perceptions, and attitudes members have about digital accessibility in the context of DT.

SRT has been applied in many recent studies from an industry perspective to understand stakeholders’ shared knowledge about a social object, such as a new technology or phenomenon, or their perceptions and attitudes toward it. Vieira and Joia (57) applied SRT to examine how IT professionals conceptualize DT and found a notable discrepancy between literature-based definitions and practitioners’ perceptions. These findings suggest that IT managers in practice struggle to integrate DT into their strategic and operational frameworks, reflecting a techno-centric and limited understanding of the concept. Na et al. (58) presented the differences among the perceptions and attitudes of two groups, care service managers and care workers, toward care robots, a technology that has relatively low adoption rates. The study presented its findings in terms of enablers for technology adoption and inhibitors in terms of resistance to new technologies.

SRT has also been used in emerging technology fields to reveal key stakeholders’ perceptions about the industrial and societal changes that technology will bring, as well as the inherent opportunities and challenges of technology-driven change. Jang et al. (59) applied SRT to understand the perceptions of managers in the financial industry to provide successful services through a new technology called chatbots. In the context of the music industry, Park and Kim (60) used SRT to explore artists’ perceptions of the opportunities and challenges that a new technology called blockchain could bring. Park and Kim (61) studied the shared perceptions of how people with disabilities want to represent their disabilities based on avatars in virtual world platforms to provide direction for barrier-free virtual world services. SRT is also ideal for exploring concepts where theoretical definitions diverge from practical understandings. Michelotto and Joia (62) revealed a gap between citizens’ perceptions of smart cities and academic definitions, reflecting conceptual inconsistencies. Similarly, this study adopts SRT as an analytical perspective to examine employees’ shared understandings and representations within the restaurant industry. Table 1 summarizes prior studies that have adopted SRT to examine stakeholders’ perceptions across various contexts.

**Table 1:** Prior research employing SRT.

Key prior research	Context	Key finings
Vieira & Joia (57)	IT professionals’ perceptions of DT	Identified a gap between literature-defined concepts and practitioner views, highlighting a techno-centric perspective.
Na et al. (58)	Elder care workers’ and managers’ perceptions of care robots	Analyzed the social representations of two key groups as service managers and care workers in care facilities, revealing contrasting perceptions and attitudes shaped by their differing roles.
Jang et al. (59)	Financial industry managers’ views on chatbot services	Comprehensively presents opportunities and challenges for managers in the Korean financial industry to understand chatbot services.
Park & Kim (61)	Avatars and disability representation in virtual worlds	Explored how people with disabilities want to express their identity through avatars in virtual worlds, providing insights for designing inclusive virtual worlds.
Michelotto & Joia (62)	Citizens’ understanding of the smart city concept	Refining the concept of smart cities as perceived by citizens and contributing to bridging the gap between academic and everyday definitions.

Franchise restaurant firms in South Korea, especially those targeting individuals in their 20s and 30s, are increasingly using kiosk devices to replace traditional in-person orders. However, there is a scarcity of studies that have probed the perceptions of employees of Korean franchise restaurant firms regarding kiosk accessibility, despite the technology’s widespread ubiquity. Consequently, this study directs its attention to older adults’ experiences of digital alienation, highlighting their status as a digitally disadvantaged group in the franchise restaurant industry. To this end, perceptions of kiosk accessibility among employees are explored, as they are the primary actors capable of directly enhancing kiosk accessibility. Therefore, this study proposes the following research questions.

RQ. How do employees of Korean franchised restaurants perceive kiosk accessibility for the older adults?

## 3 Methodology

To understand perceptions of older adult access to kiosks among employees in Korea’s franchise restaurant industry, core-periphery analysis of SRT was applied. In the methodological implementation of SRT theory, the analytical procedure proceeds in three main stages: eliciting social representations, performing content analysis, and analyzing the representation structure (63). First, we conducted in-person interviews with employees, as they are stakeholders in the decision-making process of operating and managing kiosks and improving accessibility. The interviews were transcribed, and a content analysis was then conducted on the interview data. The extracted topics were structured based on core-periphery analysis (64), reflecting employees’ understanding and perceptions of kiosk accessibility and the digital inclusion of older adults. We coded phrases that represented units of meaning. Starting with open coding, the coding process was refined through iterative discussions among the authors, during which codes within subclusters were grouped and classified into higher-level clusters. A co-occurrence matrix of the codes was then generated, and the core-periphery topics were classified based on the calculated coreness values. Furthermore, the perceptual map of the core-periphery structure was represented as a maximum tree to identify the relationships and similarities between the topics (65). Details of the data collection and analytical procedures are presented in Sections 3.1, 3.2, and 3.3.

### 3.1 Eliciting social representations of older adult users kiosk accessibility

The study conducted semi-structured, face-to-face interviews with 12 employees in the franchise restaurant industry. To arrange for the interviews, researchers first contacted the ESG management team of a franchise restaurant headquartered in South Korea. Although there are several franchised restaurants in Korea, many are global chains headquartered overseas. However, for this study, it was deemed appropriate to interview stakeholders directly involved in identifying and reflecting on the issues that arise in the industry and making decisions to improve it. In addition, it was important that the interviews were representative of Korea’s specific industry.

Employees of these franchised restaurant brands were ideal interviewees for this study because of the firms’ high kiosk adoption rates, thus making ordering through kiosks commonplace. They were also an ideal choice because of their work with governments and municipalities to provide digital literacy training and device development to improve older adult kiosk accessibility. For the researchers to conduct on-site interviews, permission was required at the manager and headquarters levels. During the data collection process, the study’s purpose and the research ethics were explained; the anonymity of participants and their companies was also guaranteed. Following these procedures, the researchers personally visited the stores where the kiosks were installed and the franchise headquarters that managed them to conduct the interviews.

Interviews were held between March and April 2024, and interviewees were at both the managerial and supervisory levels, with a minimum of 7 years and a maximum of 19 years of experience. Interviewees are the stakeholders involved in decision-making regarding the operational management of kiosks and in the process of improving kiosk accessibility. Although the sample size of 12 interviewees is small, each interviewee represents either a specific brand or a dedicated functional department within the organization. They are on the front-end, facing customers in the field and experiencing their reactions first-hand to the convenience and inconvenience of using kiosks, as well as the profit and loss of the company. Accordingly, this study incorporates perspectives from both managerial staff responsible for company-wide operations and frontline practitioners who directly engage with customers and manage individual brands or stores.

However, the number of people involved in improving kiosk accessibility at the business unit level is very limited. Furthermore, despite the proliferation of ordering through kiosks, older adult accessibility has yet to be addressed in the industry. Therefore, this study sought to understand practitioners’ perceptions of the current state of kiosk accessibility and future challenges through face-to-face interviews.

The interview questions were organized around the following: what digital accessibility and inclusion for older adults is perceived to be from the perspective of practitioners in the franchised restaurant industry, how they perceive the current state and need for kiosk accessibility for older adults, what the practical challenges are that need to be addressed when improving kiosk accessibility, how they define the scope of their customer base, and whether they perceive improving kiosk accessibility and digital inclusion for older adults as a positive possibility for their company or as a burden and concern.

Responses were recorded using a voice recording app on a smartphone with participants’ consent. The average interview lasted between 30 min and 1 h, with additional questions and answers based on the interviewee’s responses. There were no refusals or dropouts during the interviews. Interviewees’ descriptive information is presented in Table 2.

**Table 2:** Descriptive information of interviewees.

Interviewee	Business field	Profession	Core work content	Position	Years in the profession	Gender
1	Burger	Sales	Franchise operations support, POS standardization, store management supervision	Manager	16	Male
2				Assistant Manager	11	Male
3	Coffee	Promotions	Kiosk operation planning and promotional execution	Associate	8	Male
4	Special Retail/Concession	Strategic planning/Sales support	Strategic planning, and sales support for specialty stores in transportation and retail hubs	Associate	7	Male
5	Donuts	Sales	Sales management for external distribution channels	Manager	13	Male
6	Smart Store	Digital Transformation	kiosk development for smart-store digital transformation, customer management, and store operations	Manager	16	Male
7	ESG	ESG Management/Corporate Strategy	Headquarters ESG management and franchisee business operations	Team Manager	19	Female
8				Manager	13	Female
9	Design	UI/UX design	Customer-oriented design operations and brand image development	Assistant Manager	10	Male
10	Burger	Store management	Direct operation and management of a headquarters-operated franchise store	Store Manager	13	Female
11					13	Male
12				Assistant Store Manager	7	Female

### 3.2 Content analysis

Interview data were transcribed, and detailed coding was conducted to identify topics. Text segmentation of the interview content involved dividing utterances into meaningful units within paragraphs, followed by syntactic-level coding. The primary coding was administered as an open coding process, with the authors defining and categorizing each code after several rounds of discussion. In the initial round of coding, 80 codes were identified via content analysis. After identifying codes that could categorize them based on the interviews, the authors held several discussions to combine or eliminate codes if they were not mutually exclusive or could be incorporated into higher-level concepts. This process ultimately resulted in 15 codes (components). In qualitative research, data collection continues until the sample fully represents all aspects revealed in the data, and the exact number of participants or interviews required depends on the goals and purpose of the study (66). The authors determined that data saturation had been reached during the content analysis process, as no new codes emerged that were sufficiently recurrent to be incorporated into higher-level clusters.

The final identified codes were applied to code the interview data, and another coder independently coded 50 samples in the same manner to assess inter-coder reliability. The Cohen’s Kappa value for inter-coder reliability was 0.787, indicating a high level of agreement among coders (67). Once inter-coder reliability was established, the authors proceeded with independent coding, with a sample for reliability assessment included in the overall dataset. Table 3 presents the finalized 15 topics derived from the interviews.

**Table 3:** Topics in the social representation of older adults concerning kiosk accessibility.

Topic		Example
T01	Improving customer convenience	“From the customer’s perspective, accessibility simply means making the process fast and straightforward.” (Interviewee 1)
T02	Bridging technology	“AI and mobile technologies will continue to evolve, and kiosk usage will naturally decline as more advanced solutions take their place.” (Interviewee 4)
T03	Interim digital transformation	“Our ultimate goal is to eliminate kiosks, but people need time to adapt, so digital transformation must proceed step by step rather than by introducing future technologies all at once.” (Interviewee 6)
T04	Difficulty in meeting diverse demands	“From the franchisee’s perspective, the more exceptions they have to handle, such as some customers being able to use certain options while others require manual ordering, the less accessible the system becomes for them.” (Interviewee 1)
T05	It does not fit most customers	“If kiosks are designed primarily around the needs of people with disabilities or other vulnerable groups, the majority of customers may experience inconveniences compared with the existing system.” (Interviewee 2)
T06	Considering older adults	“Older people used to visit stores easily, but once kiosks were introduced, they began to wonder, ‘Why is this suddenly difficult for me?’ I believe older adults have become the real digitally excluded group.” (Interviewee 8)
T07	Balance between profit and social values	“There is a tension between social values and corporate priorities, and because companies focus on profit, social values can easily be overlooked.” (Interviewee 4)
T08	Improving franchise owners’ convenience	“From the store’s perspective, having fewer direct customer interactions will make operations much easier.” (Interviewee 12)
T09	Labor cost reduction	“Labor costs keep rising, and on holidays part-time wages increase up to 2.5 times, creating a significant financial burden.” (Interviewee 10)
T10	Reducing some choices	“If we simplify the kiosk for digitally vulnerable users—such as by reducing the number of menu items displayed—young customers who prefer seeing many options at once may feel inconvenienced, which raises concerns about reverse discrimination.” (Interviewee 7)
T11	Lacking face-to-face service	“Service is ultimately about human interaction, and some customers prefer simply speaking to a staff member rather than navigating a kiosk. Since every customer is different, kiosks cannot replace all of the services that people can provide.” (Interviewee 1)
T12	Necessary investment	“I see this as an unavoidable phenomenon. The opportunity was years ago, and the challenge is what we are dealing with now.” (Interviewee 5)
T13	Business opportunity	“If we improve accessibility and make the system more convenient for vulnerable users, it could become an opportunity for us. Older and socially disadvantaged customers are also a segment we can potentially reach.” (Interviewee 3)
T14	Organizational and social challenges	“Because we are fundamentally a food-based company rather than a digital or IT-driven organization, adapting to rapid technological change presents a significant challenge.” (Interviewee 7)
T15	Emergency or extraordinary reactions	“In reality, some customers still need specific explanations—for example, details about ingredients that may not be fully displayed or easy to find on the kiosk—which requires human assistance.” (Interviewee 3)

### 3.3 Core-periphery analysis of social representation structure

A core-periphery analysis was conducted, and topics were divided into core and peripheral components. The core-periphery model was suggested by (64) to identify the structure of network data. The core remains stable and enduring across diverse contexts, structuring the peripheral elements, whereas the peripheral elements can adapt to changing environments without undermining the core (55). The criteria for identifying core elements of social representations are categorized as symbolic value, expressive value, and associative value (55), and symbolic value requires additional research settings such as experimental designs (63). Therefore, this study focused on expressive value and associative value, conducting structural analysis primarily based on whether central topics exist more frequently than peripheral topics and whether central topics are associated with more topics than peripheral topics.

Initially, to construct the analytical matrix, the frequency with which two topics were mentioned simultaneously in the same response unit was counted, and a co-occurrence matrix was created in Excel. In this study, a response unit was defined as a sentence or paragraph that conveyed a complete idea expressed by the interviewee. The parameter salience indicates the frequency of a topic within responses (55), and summing similarity evaluates associative value (65). An inter-attribute similarity (IAS) matrix was then constructed, with each cell containing the Jaccard similarity coefficient automatically calculated by the software (68).

Core–periphery analysis was conducted in UCINET software to generate the coreness value and membership of the core and periphery components. This model identifies core and peripheral structures based on coreness, which reflects the degree to which each topic is positioned closer to the center of the network (64). Based on each element’s coreness value, topics with high coreness were classified as core elements, while the remaining topics were categorized as peripheral. The co-occurrence matrix served as the data matrix for this analysis, and the strength of connections between nodes reflects their co-occurrence values.

As a result, two topics were classified as core, and the remaining 13 were classified as peripheral in representing franchise restaurant employees’ perceptions of older adult kiosk accessibility. Each topic’s classification within the core and periphery structure is detailed in Table 4.

**Table 4:** Each topic’s core and periphery structure.

Code	Topics	Coreness	Membership
T11	Lacking face-to-face service	0.773	CORE
T15	Emergency or extraordinary reactions	0.621	
T14	Organizational and social challenges	0.083	PERIPHERY
T10	Reducing some choices	0.042	
T07	Balance between profits and social values	0.039	
T09	Labor cost reduction	0.038	
T01	Improving customer convenience	0.036	
T08	Improving franchise owners’ convenience	0.03	
T05	It does not fit most customers	0.028	
T13	Business opportunity	0.026	
T06	Considering older adults	0.025	
T03	Interim digital transformation	0.023	
T02	Bridging technology	0.017	
T04	Difficulty in meeting diverse demands	0.017	
T12	Necessary investment	0.008	

Core components are shaded on the map.

### 3.4 Mapping social representation

Maximum trees can visualize a social representation of a social group’s perceptions and can be useful for presenting results to practitioners, including frequency and relationship information, in a more intuitive way (69). To identify the relationships and connections among topics, this study mapped the associations among the 15 topics and visualized the network between the core and periphery elements as a maximum tree. This analysis is based on the computation of co-occurrence of textual data, meaning relationships among topics with similar semantics (70). In similarity analysis, the higher the sum of similarities among topics, the more closely they are related to other elements (69). Jaccard’s similarity coefficient was used to measure the strength of similarity or connection among topics (68).

The maximum tree was constructed using a nearest-neighbor algorithm, which sequentially connects two components based on their highest similarity values (63). The mapping process began by identifying the topic with the highest salience value. Subsequently, from the remaining topics, the topic with the highest similarity coefficient in the IAS matrix to any topic already included in the map was connected next. When similarity values were identical for multiple topics, the topic with the higher salience value or higher summing similarity was selected. This iterative procedure continued until all topics were incorporated into a single connected structure. Once all topics were included, one-to-one links were drawn based on coreness values to finalize the social representation map. Based on these connections, perceptions of older adult kiosk accessibility held by practitioners in the franchised restaurant industry were visualized as a maximal tree (71).

## 4 Results

**Figure 1:**
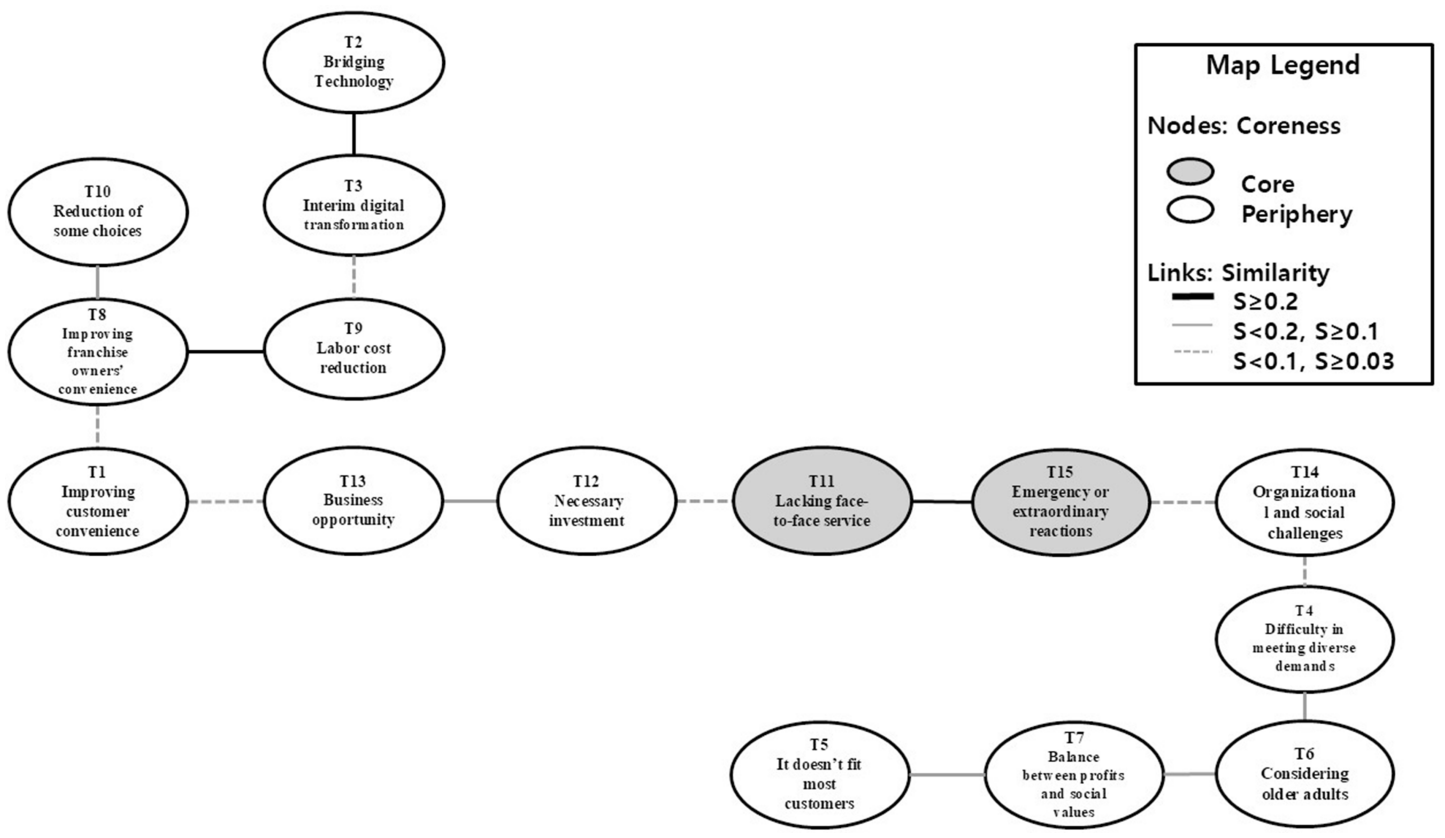
Social representation map of employee perceptions regarding older adult kiosk accessibility.

Content analysis results indicate 15 components of older adult kiosk accessibility, as perceived by employees in Korea’s franchise restaurant industry. The core-periphery analysis shows that two of the 15 topics were core (concentration = 0.990), and the remaining 13 were periphery (Table 4). The core components are Lacking face-to-face service (T11) and Emergency or extraordinary reactions (T15). The social representation map in Figure 1 visualizes topics and links of the overall structure of social representation by employees. The dark-shaded components are core topics, and thicker lines indicate greater similarity among topics. Each node is labeled with its topic number and corresponding topic name, as identified through the content analysis. The strength of connections between nodes is based on co-occurrence values (72). The map legend reflects these visual elements, indicating core versus peripheral topics and representing connection strength.

## 5 Discussion and conclusion

The social representation map revealed employees’ perceptions and attitudes regarding older adult kiosk accessibility and digital inclusion in Korea’s franchise restaurant industry (Figure 1). Core topics constitute a shared understanding among stakeholders and consistently emerge across various contexts in core–periphery analysis, thereby influencing the structuring of peripheral topics (55). In this study, the lack of face-to-face service (T11) and emergencies or extraordinary reactions (T15) emerged as core themes, shaping employees’ understanding of kiosk accessibility for older adult users. This relationship can also be interpreted in the context of Korean service culture. Previous studies have shown that older adult often experience social anxiety when using kiosks, particularly when they feel observed by others (7). This anxiety often prompts them to seek interpersonal assistance from staff as a coping strategy.

Emergencies and extraordinary reactions (T15) continue to occur, and the lack of face-to-face service (T11) remains a concern. These themes highlight business opportunities (T13) that may arise when operators respond strategically to changes in customer interaction patterns driven by the digitization of in-person ordering. However, they also give rise to organizational and social challenges (T14) as firms navigate diverse stakeholder needs and on-site operational demands. Sections 5.1 and 5.2 further explain how practitioners perceive the relationships between the core themes and associated peripheral topics regarding older adult kiosk accessibility.

### 5.1 Business opportunities

**Figure 2:**
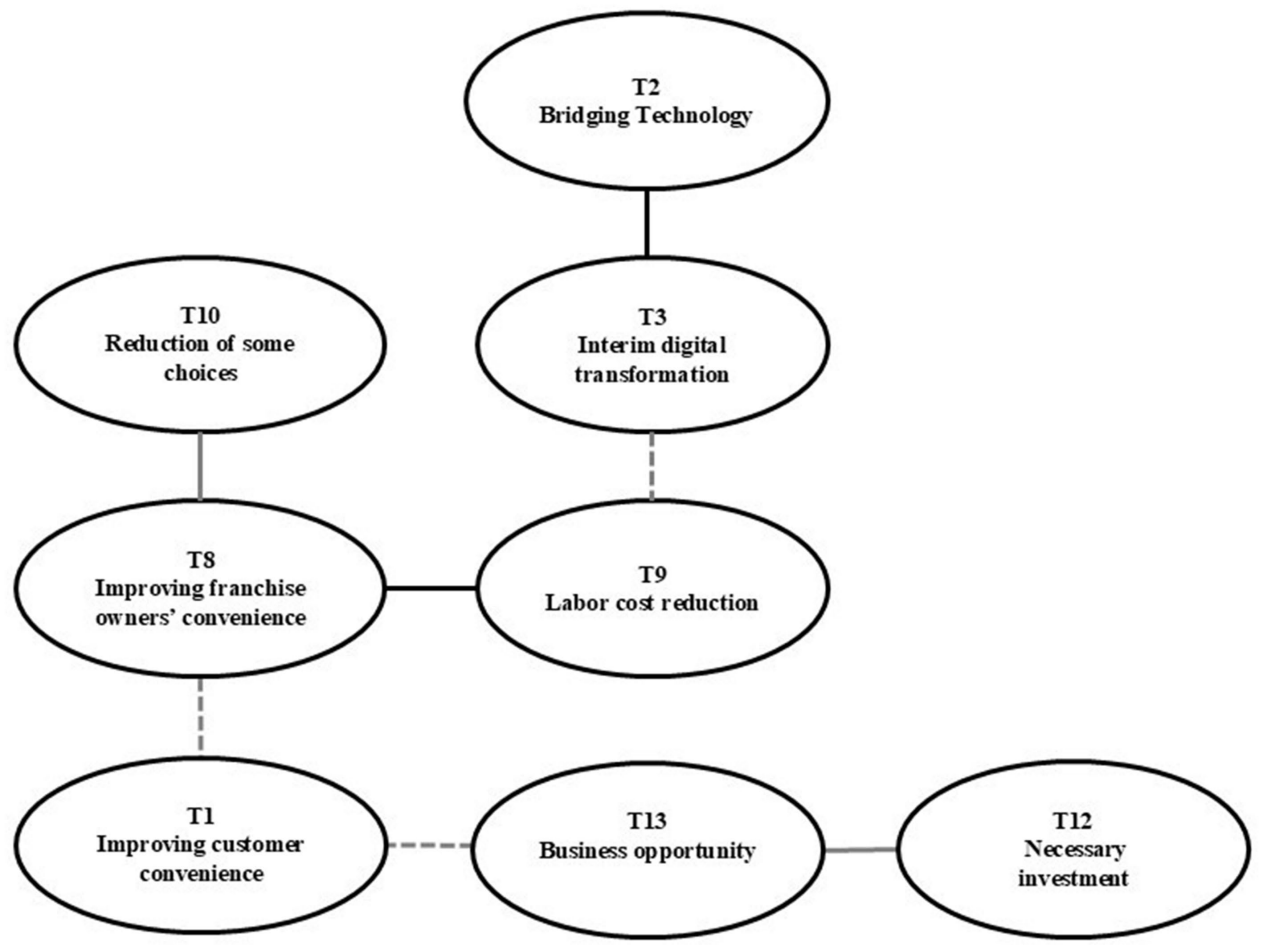
Business opportunities.

The relationship demonstrates the business opportunities (T13) concerning employees’ awareness of older adult kiosk accessibility (Figure 2). While kiosks cannot completely replace face–to-face service or extraordinary service, interviewees perceived that making ordering more accessible before other brands did so could lead to increased Business opportunities (T13). Furthermore, many companies expect DT to drive value creation and business opportunities beyond organizational operations (30). For some franchisees, the consensus was that the service they provide determines whether customers will return.

Employees perceived that efforts to include the digitally disadvantaged were somewhat mandated by social values, as society was demanding digital inclusion and competitors were doing the same. In addition, kiosks have already been commercialized in the franchise restaurant industry, and significant investments have already been made in the process (49). There was, therefore, a positive perception that if companies were proactive by making the Necessary investments (T12), it could also connect to Business opportunities (T13), such as expanding customer reach or enhancing brand image. In this relationship, “Necessary investments” (T12) refer to actions that firms deem essential for sustaining their digital transformation efforts. In contrast, “Business opportunities” (T13) refer to the broader, long-term value that can result from these investments.

Furthermore, Business opportunities (T13) perceived by employees held largely two perspectives concerning Improving customer convenience (T1) and improving franchise owners’ convenience (T8). In terms of the former, franchise headquarters employees and field managers expected that simplifying or standardizing kiosks, thereby rendering them more accessible, would make it easier for more customers to access what they want. Kiosk-mediated customer-technology interactions are increasing in a range industries, and kiosk service quality can influence consumer behavior by increasing customer satisfaction and loyalty (73).

However, in terms of Business opportunities (T13), it was the franchise owner’s perspective rather than the customer’s that showed more connections to the themes. The interviewees were either headquarters-based or franchisees managing headquarters-owned stores. From the perspectives of headquarters-based employees, customers included not only those who consume the company’s products but also franchise owners, the latter of whom were also deemed important customers. Improving franchise owners’ convenience (T8) is very strongly linked to Labor cost reduction (T9), which can be interpreted as a positive perception of operational efficiency through labor replacement and labor cost savings through kiosks. Previous studies have already demonstrated that digital transformation can increase the efficiency of a company’s labor investment by significantly curbing overinvestment in labor and over-hiring (74). Employees expected that kiosks’ increased senior accessibility would allow more customers to use them to place their own orders, which would reduce labor costs and boost profits.

In addition, Labor cost reduction (T9) is the bridge between Improving franchise owners’ convenience (T8) and Interim digital transformation (T3). Interim digital transformation (T3) is defined as the perception among employees that kiosks are transitional technologies adopted while the industry moves toward more advanced, personalized digital systems. Employees expect to increase operational efficiency through DT, including kiosks. As one participant reported, “I think older adults are getting over the barrier of kiosks being everywhere they go, but there are still many people who are angry, and just want an employee to place their order for them. To utilize manpower more, we have part-time workers who clean outside the kitchen and also help older adults use the kiosks, but it’s difficult to do that in stores with low sales, so we need to increase kiosk utilization (Interviewee 10).” Therefore, employees believe that increased kiosk accessibility can lead to better Business opportunities (T13) by improving the convenience of franchisees, who, in turn, play an important role in the franchise business.

However, for improved kiosk accessibility to improve franchise owners’ convenience, it must be considered alongside Reducing some choices (T10). In the words of Interviewee 5: “Although improving accessibility for older adults can simplify ordering at the kiosk, the major problem is that if a customer complains that they did not receive a discount or credit after paying through the kiosk, it is a process that can only be handled face-to-face by a manager who responds entirely on-site.” In other words, there is a concern among employees that increasing older adult accessibility may lead to Reducing some choices (T10), yet increasing accessibility could improve convenience for customers (T1) and franchise owners (T8) when done in a way that does not generate such complaints.

Another strongly connected topic is Bridging technology (T2) and Interim digital transformation (T3). DT is not just about applying digital technologies to existing products and services or replacing face-to-face services with digital tools, but rather, it affects a set of business operations, organizational structures, changes in stakeholder relationships, and customer value creation processes (75). Among interviewees, kiosks were perceived as moving toward digitizing the ordering process, from production to ordering to delivering the customer’s receipt and reordering. Therefore, including older adults in the digital realm by making kiosks more accessible to them at the ordering stage is vital for retaining them as customers for the rest of the DT journey, from product pickup to return visits. Simultaneously, however, employees recognized that kiosks were not the final solution, but rather an interim bridging technology. It was believed that, in the future, they would need to prepare to move toward personalized devices like mobile phones or new technologies like AI. Responding to and addressing these changes well could lead to future Business opportunities (T13).

### 5.2 Organizational and social challenges

**Figure 3:**
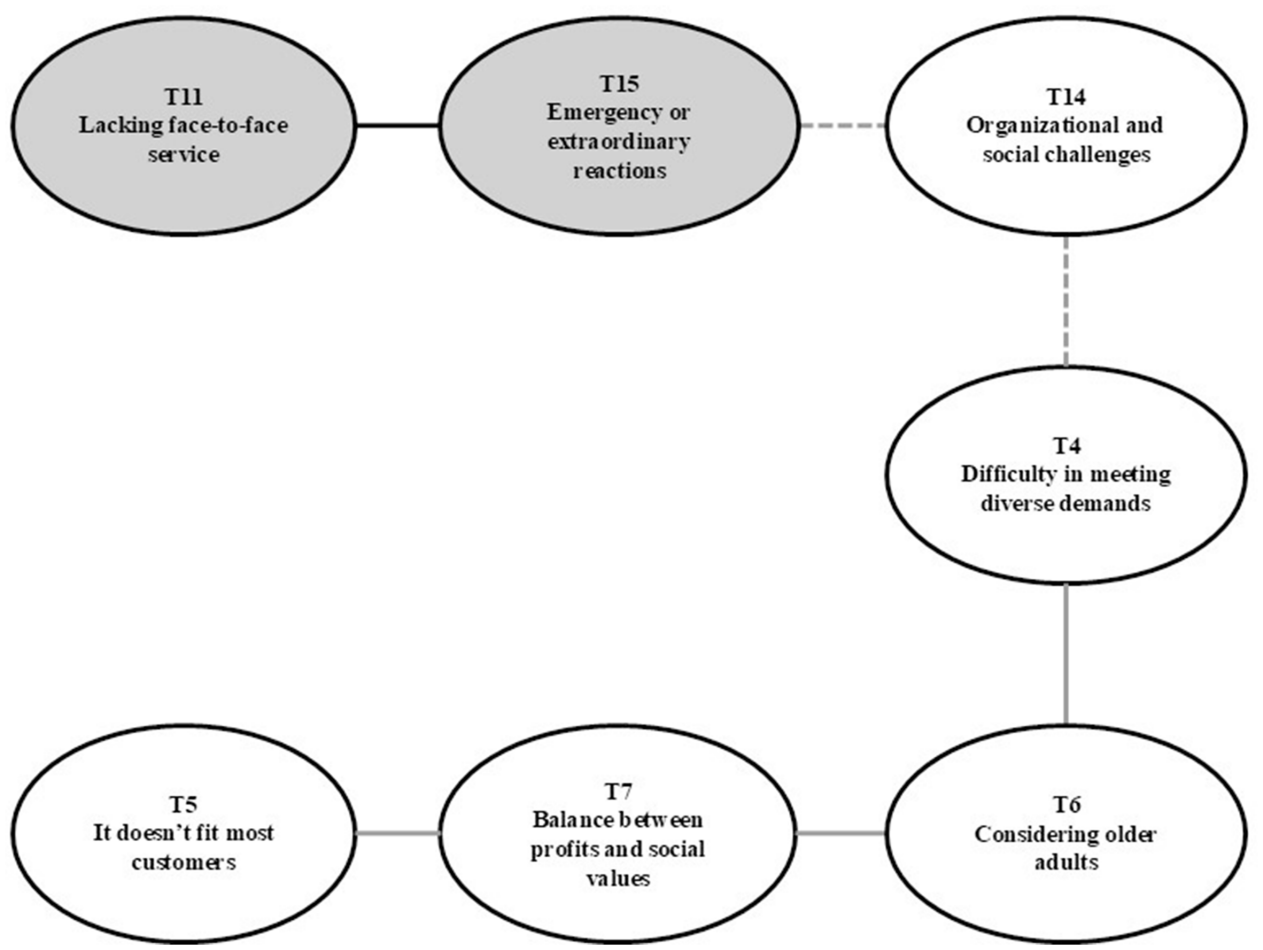
Organizational and social challenges.

The second relationship reflects the challenges concerning the organizational and social aspects of employees’ awareness of older adult kiosk accessibility (Figure 3). While improving access could lead to Business opportunities (T13), challenges, including difficulties in meeting diverse demands and maintaining a balance between profits and social values, were identified as implementation barriers. With this digital shift of using kiosks already underway, interviewees were significantly concerned that one device cannot replace all Face-to-face services (T11). Moreover, they worried that kiosks cannot constitute a complete solution for emergencies or responding to extraordinary customer reactions from older adult customers. While the central attribute of automation-driven operational efficiency and improved customer experience could lead to opportunities depending on how DT is achieved, interviewees perceived that the current kiosk limitations also presented pressing challenges.

In this context, employees in the franchised restaurant industry perceive themselves as facing organizational and social challenges (T14). As one participant said, “The franchise restaurant industry is not a digital-based industry, it’s a food-based company, so it’s not an organization that can change as fast as the IT sector, so making kiosks accessible for older adults is an organizational challenge” (Interviewee 7). For restaurant companies, ESG management has been considered regarding food safety, environmental issues, and the ethical governance operations of managers (76). Normally, the way restaurant firms have fulfilled their social responsibility has been by providing quality food, improving product quality for customers, and business value for owners (77). However, given that much of the restaurant industry has gone digital, accessibility for older adult customers has been perceived as another important organizational and social challenge for companies.

Employees believe that improving older adult kiosk accessibility is realized by streamlining and making the kiosk steps easier. However, employees also face difficulties concerning meeting diverse demands (T4). These varied needs include those of the general customers, that is, those excluded from the digitally disadvantaged, and the demands on the headquarters of franchise owners, who must respond on-site to complaints related to kiosks. Among the challenges registered by employees, is that considering the general customer only can exclude older adults. Conversely, considering older adult accessibility can inconvenience other customers or franchise owners. As such, bridging this gap is both imperative and a key challenge.

There is a perception among employees that making kiosks more accessible is a way to considers older adult (T6). The older population is among the most vulnerable when it comes to technology use, and seniors can have negative emotional reactions to using kiosks due to physical, cognitive, and psychological factors (7). However, there is also a perception that kiosks that take older adults into consideration are unfit for most customers (T5). These two opposing concerns are connected by the perception of balancing profits and social value (T7).

While the target customers that contribute directly to the restaurant’s revenue comprise younger generations, employees perceive making kiosks more accessible to seniors is something that contributes mostly to revenue and is not beneficial to most customers. As one participant noted, “We should try to make our kiosks more accessible to older adults, who are the most digitally disadvantaged in society, but we are a private company, not a public one. It costs money, and I do not think it will have a direct impact on sales in the short term” (Interviewee 6). Another said, “However, we are seeing fewer births and an older population. I do not think digital inclusion is a short-term benefit for the company, but it’s a long-term challenge” (Interviewee 7). Companies undergoing DT are struggling to achieve the dual goals of reducing costs through automation and increasing revenue through improved customer experience (75). For employees who must consider the company’s bottom line, the fact that improving the accessibility of kiosks is driven by social value rather than company revenue poses a challenge in implementing change.

### 5.3 Key findings

The key finding of this study is its identification of how corporate operators in Korean franchise restaurant companies understand older adult kiosk accessibility within the context of digital transformation. Content analysis and core–periphery analysis revealed the structure of these perceptions, showing that employees associated kiosk accessibility for older adults with business opportunities as well as organizational and social challenges. Employees believed that while current kiosks could not replace all face-to-face services or cater to on-site emergencies or extraordinary needs, they could see more business opportunities with kiosks if they could provide a more detailed customer experience based on customer data during DT. They also expected improved customer convenience, greater convenience for franchisees, and lower labor costs by making kiosks more accessible to seniors. Furthermore, as part of their planned service-wide DT, attracting and retaining an increasingly large share of the older adult demographic as customers was seen as a business opportunity.

Although the opportunities presented by making kiosks more accessible to older adults were considered primarily from a business perspective, the main concerns were seen as organizational and social challenges: current kiosks are insufficient in fully replacing in-person services or responding to on-site needs, which can lead to increased workloads for managers. Employees recognized that improving older adult kiosk accessibility is a social need in the context of digital inclusion. However, they also noted that companies face an organizational challenge of reconciling the needs of multiple stakeholders, including the profit-driven objectives of the business and the average customer, who has already become accustomed to using kiosks as they exist now. In this situation, regarding considering older adult kiosk accessibility, there is a concern that designing kiosks to make them easier to use for older adults will not work for most customers.

The study also found that a major theme bridging these topics is the concern to balance profit and social value. Private companies operate to maximize business profits. Simultaneously, companies are not simply independent businesses, but interdependent enterprises that must survive within social and ecological networks (78). Therefore, when companies conduct sustainable and inclusive business, they can generate social benefits and contribute to the quality of many people (22). Given this study’s findings, emphasizing efforts to improve older adult kiosk accessibility solely as an operator obligation may increase the burden on businesses. Instead, recognizing kiosk accessibility as a long-term benefit to businesses by improving the customer experience and expanding their customer base is more appropriate. Consequently, this approach can contribute to achieving corporate sustainability and enhancing social value through digital inclusion.

## 6 Implications and limitations

### 6.1 Theoretical implications

This study offers an important theoretical contribution by applying Social Representation Theory (SRT) to explore the perceptions of employees in the restaurant industry, thereby extending SRT’s scope as an analytical framework in industry contexts. First, this study applies SRT to the franchise restaurant industry, offering a conceptual framework for analyzing employees’ shared perceptions of digital accessibility. Through a core-periphery analysis, the study identifies the cognitive structure through which franchise operators interpret kiosk accessibility for older adults. Furthermore, the topics and relationships revealed in the social representation map suggest factors for future research to explore.

Secondly, this study addresses a gap in research on the industrial and societal changes caused by technological advancements. It investigates kiosks that have already been integrated into everyday service settings, but which still pose accessibility challenges. These challenges cannot be adequately explained by traditional concepts addressed in prior kiosk research, such as technology adoption (79) or resistance to innovation (80). By examining the perceptions of practitioners who operate and manage kiosk-based services, the study provides theoretical insight into why accessibility for older adults is not prioritized enough in the private sector. It also clarifies the structural opportunities and constraints associated with improving accessibility.

Third, the study reframes digital accessibility as a socially constructed organizational issue rather than an individual-level usability problem or device-centered design concern. This conceptual shift enriches theoretical discussions of digital inclusion, positioning accessibility as a component of organizational strategy and digital transformation rather than a technical challenge or matter of individual digital literacy.

### 6.2 Practical implications

The research has practical implications for practitioners in organizations undergoing DT, as well as for policymakers. The study found that, in the context of kiosk usage, the lack of face-to-face service and the difficulty of responding to emergencies or extraordinary situations were core topics for employees. These results suggest that kiosks cannot entirely replace in-person service, either for customers or employees. Recommendations include the following. One strategy that operators can adopt is considering the balance between services provided by technology and humans. Another is that the kiosk’s design should reflect the customer’s perspective to ensure its universal accessibility, for both major and minor users. Third, operators can consider retaining human employees for consumers who need to adapt to new technologies and improve their literacy during the DT. If companies preserve human assistance for customers who experience barriers to kiosk usage, they may increase their customer base.

In contrast to previous studies that focused on corporate social obligations regarding digital accessibility among seniors, this study reveals that employees perceive enhancing accessibility as a business opportunity. Improving access not only enhances customer convenience but also increases operational efficiency, leading to benefits such as enhanced owner convenience and reduced labor costs. Indeed, sustainable operations can positively impact a company’s empirical performance and corporate value (81). Ensuring that digital technology does not exclude certain customers can be considered a strategic approach for firms to expand their market reach and enhance their overall offerings, particularly by catering to the increasingly socially engaged older adult customer.

Furthermore, this research holds implications for policymaking regarding seniors’ digital inclusion. The digital divide, which, internationally, separates the population into those with access to ICT technologies and those without it, has shaped how public and subsidized services are provided to achieve universal service and access to ICT technologies (10). In addition, establishing standards for kiosks is vital, as is considering whether these standards are created from the user’s perspective or reflect corporate interests (82). South Korea has introduced digital accessibility standards for senior-friendly kiosks, recommending user interface (UI) and user experience (UX) designs, as well as information structures, that are tailored to the needs and characteristics of older adults (83). Additionally, a partial amendment to the Act on the Prohibition of Discrimination Against Persons with Disabilities was passed in 2025. This amendment requires the installation of barrier-free kiosks that meet the Ministry of Science and ICT’s verification standards, and features such as voice guidance are mandated to enhance accessibility (84). While these policies reflect user needs and establish important regulatory expectations, it is crucial to understand the perspectives of practitioners in industrial settings, given that businesses are ultimately responsible for implementing these standards.

Integrating these discussions suggests that the efforts of policymakers to improve digital accessibility for older adults are not in conflict with the interests of corporate operators. Improving digital accessibility often only requires modest adjustments. However, companies that overlook such efforts risk long-term legal, reputational and commercial consequences for failing to meet the needs of users with disabilities and older adults, which ultimately affects business success (85). Accordingly, policymakers should incorporate the needs of older users into kiosk standards while encouraging positive incentives for corporations, such as expanding customer reach and advancing digital transformation. At the same time, policy design should address the practical challenges operators face, such as financial and operational burdens, to ensure that accessibility improvements are feasible and sustainable in the private sector.

Older adult users must make a personal commitment to actively engage in education and experiences to improve their digital literacy. As this study reveals, employees in the franchised restaurant industry are preparing for a broader DT across services. As employees’ perceptions show, kiosks are a bridging technology to newer technologies such as personalization devices and AI. Therefore, older adult users will need to learn and adapt to the digital technologies that will continue to be an inevitable part of their daily lives. The private and public sectors alike should explore ways to collaborate to support these efforts.

### 6.3 Limitations and future research

This study has several limitations and, as such, points to additional areas for future research. First, this study targeted a specific franchise restaurant industry in South Korea. Since South Korea is a rapidly aging and highly digitalized society, Korea has a high rate of kiosk adoption in both the public and private sectors. This situation may differ from those of other countries. Therefore, future research could examine cases from different countries or industries, particularly in other aging societies or service sectors undergoing digital transformation. This would help to assess the generalizability and contextual specificity of the findings.

In this study, interviewees totaled 12, which is representative of professionals with sufficient work experience, but is limited by the small number of participants. If a larger number of interviewees were recruited, then a richer understanding of employee perceptions could be expected. In addition, kiosks are but one example of DT; as such, companies may have different sectors, rates, and plans for DT. Analyzing the perceptions of C-suite executives, who are decision-makers within companies, or the differences in how the public and private sectors address this issue could also inform future research. Such follow-up efforts would provide a clearer understanding of how to ensure that technology does not exclude certain social groups during the DT process, and that its benefits are accessible to all. Furthermore, building on the managerial and policy implications of this study, future research could empirically evaluate actionable strategies, such as staff training programs for assisting older adults, co-design initiatives with older adults, or incentive structures that promote inclusive design innovation.

Finally, the core–periphery analysis of social representations offers an alternative method for industry practitioners to investigate digital accessibility. This approach can address the limitations of quantitative methods, wherein conceptual structures may be influenced by methodological constraints (63, 86). Therefore, integrating quantitative approaches with the grounded analytical procedures used in this study could expand the methodological framework for examining practitioners’ shared perceptions in future research.

## Data Availability

The raw data supporting the conclusions of this article will be made available by the authors, without undue reservation.
